# Contribution of Resveratrol on Periodontal Disease Control and Treatment: A Systematic Review

**DOI:** 10.3290/j.ohpd.c_2752

**Published:** 2026-07-01

**Authors:** Octave Nadile Bandiaky, Camille Bechina, Marie Vanden Berghe, Assem Soueidan, Samuel Serisier, Xavier Struillou

**Affiliations:** a Octave Nadile Bandiaky DDS, PhD. Nantes Université, Oniris, Univ Angers, CHU Nantes, INSERM, Regenerative Medicine and Skeleton, RMeS, UMR 1229, F-44000 Nantes, France. Screened and selected articles for the systematic review, contributed to the acquisition and interpretation of the data, co-wrote the manuscript, read and approved the final version.; b Camille Bechina Assistant professor. Departement of periodontology. UIC odontology 11. Nantes Université, Oniris, Univ Angers, CHU Nantes, INSERM, Regenerative Medicine and Skeleton, RMeS, UMR 1229, F-44000 Nantes, France. Contributed to the acquisition and interpretation of the data, co-wrote the manuscript, read and approved the final version.; c Marie Vanden Berghe DDS. Department of periodontology, CHU de Nantes, Nantes Université. Screened and selected articles for the systematic review, contributed to data interpretation and manuscript writing, read and approved the final version.; d Assem Soueidan Professor. Department of periodontology. UIC odontology 11. Nantes Université, Oniris, Univ Angers, CHU Nantes, INSERM, Regenerative Medicine and Skeleton, RMeS, UMR 1229, F-44000 Nantes, France. Contributed to critical proofreading and scientific discussion, read and approved the manuscript.; e Samuel Serisier Associate professor. Department of oral biology. Nantes Université, Oniris, Univ Angers, CHU Nantes, INSERM, Regenerative Medicine and Skeleton, RMeS, UMR 1229, F-44000 Nantes, France. Screened and selected articles for the systematic review, contributed to the acquisition and interpretation of the data, co-supervised manuscript writing, read and approved the final version.; f Xavier Struillou Associate professor. Department of periodontology. UIC odontology 11. Nantes Université, Oniris, Univ Angers, CHU Nantes, INSERM, Regenerative Medicine and Skeleton, RMeS, UMR 1229, F-44000 Nantes, France. Contributed to the acquisition and interpretation of the data, co-supervised manuscript writing, read and approved the final version.

**Keywords:** clinical study, periodontal disease, preclinical study, resveratrol, 3, 5, 4’ – trihydroxystilbene

## Abstract

**Purpose:**

Resveratrol, a natural polyphenol with anti-inflammatory and antioxidant properties, has been proposed as an adjunctive agent in periodontal therapy. This systematic review aimed to evaluate the effectiveness of resveratrol in controlling periodontal inflammation and alveolar bone loss in preclinical and clinical studies.

**Methods and Materials:**

A systematic review was conducted following PRISMA 2020 guidelines. Electronic databases (PubMed/MEDLINE, Cochrane Library, Lilacs, Wiley, ScienceDirect) were searched up to October 2025, complemented by manual searches of periodontal journals. Eligible studies included *in vivo* animal experiments and randomised clinical trials assessing the impact of resveratrol on periodontal outcomes. Three reviewers independently performed study screening, data extraction, and risk-of-bias assessment using SYRCLE and Cochrane tools.

**Results:**

Twenty-two studies met the inclusion criteria, including 16 preclinical and 6 clinical studies. In animal models, resveratrol consistently reduced alveolar bone loss (7.09% to 60.60%) and improved inflammatory and oxidative stress markers. Clinical trials reported variable but generally positive effects, including reductions in probing pocket depth, bleeding index, and plaque index, and improvements in clinical attachment level. Several studies also showed decreased systemic or local inflammatory cytokines. Clinical evidence remains limited by small sample sizes, methodological heterogeneity, and short follow-up periods. No clinical study evaluated radiographic bone loss.

**Conclusion:**

Preclinical studies suggest that resveratrol has anti-inflammatory, antioxidant and bone-protective effects, but clinical evidence is limited and heterogeneous. Resveratrol may be considered as an adjunct to nonsurgical periodontal therapy, particularly in aggressive periodontitis but further well-designed trials are needed to determine optimal dosage, duration, and delivery route to support its potential as a complementary therapy for periodontitis

Periodontal diseases are intricate infectious conditions characterised by inflammation. They manifest through various clinical symptoms and signs, such as periodontal pocket formation, gingival recession, attachment loss, alveolar bone loss, and tooth mobility, often clinically detectable or subclinical inflammation and spontaneous or induced gingival bleeding. These conditions can lead to tooth loss.^8,32^ The classification of Chicago 2017 categorises periodontitis by severity into Stages I to IV. Moderate periodontitis (Stage II) involves 3–4 mm of attachment loss and 15–30% bone loss, while severe periodontitis (Stages III or IV) shows at least 5 mm of attachment loss and significant bone loss. Stage IV is the most advanced, featuring major tissue damage and requiring complex treatment. Beyond clinical staging, recent advances emphasise a biological definition of periodontal diseases based on host-response mechanisms and molecular diagnostics, which may improve disease classification and personalised treatment approaches.^4,30^

According to the World Health Organization (WHO), severe periodontal diseases affect approximately 19% of adults globally. Trindade et al reported that between 2011 and 2020, periodontitis affected about 62% of dentate adults, with severe periodontitis affecting 23.6%.^40^ The pathogenesis of periodontitis arises from complex interactions between the oral biofilm and the host’s immune response, leading to microbial dysbiosis and inflammatory dysregulation. Plaque accumulation facilitates the growth of Gram-negative anaerobic bacteria such as *Aggregatibacter actinomycetemcomitans* (Aa) and *Porphyromonas gingivalis* (Pg), which in turn recruit and infiltrate inflammatory cells.

Current treatment approaches for periodontitis begin with rigorous plaque control by the patient, followed by the reduction of microbial load through nonsurgical or surgical removal of dental plaque. While targeting periodontal pathogens typically halts disease progression, outcomes are not always as successful as desired. This has led researchers to explore adjuncts to modulate the inflammatory response.^22^ Among these, plant-derived polyphenols have emerged as promising candidates for periodontal therapy. Furthermore, dietary antioxidants may play a role in mitigating oxidative stress associated with periodontal tissue damage.^6,42^

In this context, resveratrol (3,5,4’-trihydroxystilbene), a polyphenol belonging to the stilbene class, has gained significant attention for its potential to modulate inflammatory responses.^31^ Produced by plants in response to stress conditions such as microbial infections, resveratrol is found in foods like grape skins, peanuts, pistachios, blueberries, cranberries, and red wine. Known for its diverse biological effects – including antimicrobial, anti-carcinogenic, cardioprotective, neuroprotective, and diabetes-metabolic control properties – resveratrol has been studied for its impact on periodontal disease.^15^ For instance, Andrade et al demonstrated that resveratrol shows promise in controlling periodontal disease in preclinical studies, notably by reducing alveolar bone loss.^1^ They concluded that resveratrol may enhance periodontal health through its anti-inflammatory and antioxidant effects. Similarly, Inchingolo et al reviewed the impact of phenolic compounds (including resveratrol, curcumin, and quercetin) on bone metabolism and suggested these compounds positively influence bone health and could be useful adjuncts in periodontitis treatment.^19^ Mizutani et al also demonstrated that adjunctive use of resveratrol improves periodontal parameters in patients with type 2 diabetes.^25^ We hypothesised that resveratrol, due to its anti-inflammatory and antioxidant properties, would reduce alveolar bone loss and improve clinical periodontal parameters in both preclinical and clinical settings. To update this bibliographic data, this systematic review aims to assess the scientific literature on the benefits of resveratrol as a therapeutic or preventive agent for periodontitis, including preclinical animal research and human clinical trials.

## METHODS AND MATERIALS

The review protocol was registered in PROSPERO under the number CRD42022247114. The systematic review was conducted in accordance with the PRISMA 2020 (Preferred Reporting Items for Systematic Reviews and Meta-Analyses) guidelines.^29^ The PICOS (Population, Intervention, Comparison, Outcomes and Study) framework was used to define article selection criteria. Only studies meeting the following criteria were retained: Population (P) was mice or rats for preclinical studies; patients with periodontitis in clinical studies. Intervention (I) involved the use of resveratrol alone or as a therapeutic adjuvant in the treatment of experimental periodontitis in animals or in the treatment of periodontitis in human samples. Comparison (C) of periodontal parameters was made between the test (resveratrol) and control (placebo) groups. Primary outcome (O) was reduction of alveolar bone loss (ABL). Secondary outcomes were changes in plaque level, inflammatory cytokine expression and production, the total antioxidant capacity (TAC), bleeding index (BI), clinical attachment level (CAL), probing pocket depth (PPD), and adverse effects. *In vivo* (animals and humans) studies (S) using resveratrol as a single therapy or in addition to an initial periodontal treatment were included. Studies interested in the systemic effects of resveratrol, using derivatives of resveratrol or red wine, without control groups, reviews, chapters, theses, conferences, editorials, as well as *in vitro* studies, were excluded.

### Search Strategy

Original articles were searched using electronic databases (Medline, Cochrane Library, Lilacs, Wiley and ScienceDirect) until October 2025. Filters were applied to each database to exclude studies that were irrelevant or outside the scope of our systematic review, in line with the objectives of our research. Electronic searches were completed by an additional hand-search performed for the *Journal of Clinical Periodontology*, the *Journal of Dental Research*, the *Journal of Periodontal Research* and the *Journal of Periodontology* to identify relevant articles in the field. The following medical subject heading (MeSH) terms and keywords were used: (“resveratrol” OR “3,4,5 trihydroxystilbene”), AND (“periodontal disease” OR “periodontitis” OR «gingivitis”). Clearly, each database was queried using a specific equation based on these terms and keywords. This approach allowed us to ensure the comprehensiveness of our bibliographic search. Only English articles were included, and no publication dates or publication status restrictions were imposed.

### Study Selection and Data Collection

**Fig 1:**
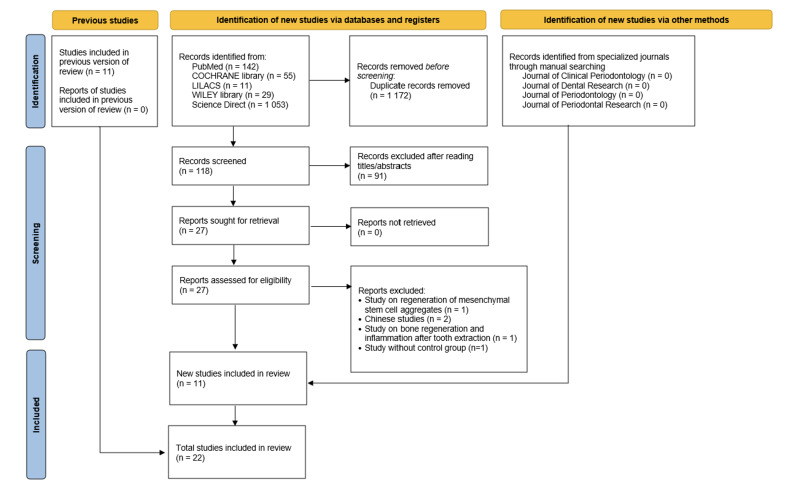
PRISMA flow diagram.

For the selection of studies, three investigators (ONB, MVB, SS) screened the titles and the abstracts of the publications in an unblended, standardised manner based on determined eligibility criteria. The second phase consisted of assessing all the articles by the same investigators to determine the eligibility of the study. The selection process was recorded in detail in the PRISMA 2020 flow diagram for the updated systematic review (Fig 1). Two study groups were established according to the type of study (preclinical studies or clinical studies). For each group, the characteristics of the study were extracted independently by the same investigators and recorded. The data were compared for accuracy, and any discrepancies were discussed and resolved by consensus. Concerning pre-clinical studies, the following data were: (1) author (year of publication); (2) animal model and study groups; (3) periodontitis induction protocol; (4) time of periodontitis induction; (5) resveratrol treatment with dose and route of administration; (7) main outcomes. The bone loss data for both the treated and untreated (control) groups were collected to calculate the relative reduction attributed to the treatment. Absolute values were either taken directly from the original tables or estimated from figures in the reviewed articles. Concerning clinical studies, following data were: (1) author (year of publication); (2) patients included (sex); (3) study design; (4) Clinical and biochemical measurements; (5) characteristic of groups (n/Group); (6) characteristic of periodontitis; (7) type of treatment (resveratrol dose and duration and periodontal therapy) and (8) main outcomes. For all groups, if one of these data points was not reported in the table, it means that information was not mentioned by the authors.

### Risk of Bias in Included Studies

Risk of bias was independently assessed by three reviewers (ONB, MVB, SS) in a blinded manner using SYRCLE’s Risk of Bias tool for animal intervention studies and the Cochrane Risk of Bias tool for randomised controlled trials, in accordance with the Cochrane Handbook.

The following domains were evaluated: selection bias (random sequence generation and allocation concealment), performance bias (blinding of participants and personnel), detection bias (blinding of outcome assessment), attrition bias (incomplete outcome data), reporting bias (selective reporting), and other sources of bias. Each domain was judged as presenting a low, unclear, or high risk of bias.

For SYRCLE’s tool, several items were frequently rated as “unclear” due to insufficient reporting of methodological details. To better characterise reporting quality, two additional descriptive items (“mention of randomisation” and “mention of blinding”) were recorded; however, these were not included in the standard risk-of-bias judgment.

When the number of animals reported in the Methods section corresponded to that in the Results section, no exclusion of animals was assumed. Disagreements between reviewers were resolved by consensus.

## RESULTS

The initial search of all sources yielded 1.290 records. Of these, 1.172 duplicated studies were removed using the reference manager EndNote®. Among the 118 articles screened, 91 were excluded after reading titles and/or abstracts. All reports were retrieved. Among the 27 reports assessed for eligibility, 16 reports were excluded (1 because it was a study on regeneration of mesenchymal stem cell aggregates, 2 articles were written in Chinese, 1 because it was a study on bone regeneration and inflammation after tooth extraction, 1 study without a control group and 11 *in vitro* studies). Eleven studies^11,16,18,20,23,26–28,41,43,44^ met the inclusion criteria and 11 supplementary studies^3,7,9,10,12–14,17,35,39,45^ identified in a previous systematic review and meta-analysis 1 were included for a total of 22 studies. These 11 articles were also identified during our initial bibliographic search. They were therefore counted separately in the article selection process. The selection process has been detailed in the attached PRISMA flowchart (Fig 1).

### Study Characteristics

Among the 22 studies included, 16 were pre-clinical studies in animal models, and 6 were clinical studies in humans.

Table 1 presents the characteristics of preclinical studies. Ten studies used Wistar rats as an experimental model,^7,10–14,27,35,39,41^ three used Sprague-Dawley rats,^3,9,16^ and three additional articles used C57BL/6J and C57BLKS/J wild-type mice.^17,18,45^ All the articles described the use of a ligature protocol, employing silk, cotton, or elastic ligatures around the selected teeth. However, Bhattarai et al and Zhen et al went further by also administering lipopolysaccharide (LPS) and a medium containing *Porphyromonas gingivalis*, respectively.^3,45^ Duration of induced periodontitis ranged from 8 to 30 days, and in half of the studies, experimental periodontitis was induced at the beginning of the experiment. Resveratrol administration route was mostly via gavage,^9–14,27,35^ but 5 studies used normal oral administration,^16,39,41^ intraperitoneal injection^17,18^ or subcutaneous injection.^3^ Doses varied from 5 mg/kg to 25 mg/kg of body weight and the duration of treatment varied from 7 to 140 days. Only Ikeda et al used a single dose of resveratrol corresponding to 0.001% (w/w) of the body weight.^17^ Five studies assessed the effects of resveratrol on periodontal disease (PD) in conjunction with other conditions, including arthritis, smoke inhalation, diabetes, or following ovariectomy surgery.^11–13,27,35^ In 10 studies, ABL was assessed through morphometric analysis by measuring the distance from the cementoenamel junction to the bone crest in methylene blue-stained samples; although other studies evaluated ABL in haematoxylin and eosin-stained specimens, using micro-computed tomography (micro-CT) or by radiographic examination.

**Table 1:** Main features of the selected preclinical studies

Author (year of publication)	Animal model and study groups (n/Group)	PD induction	Time of PD induction	Resveratrol treatment	Main findings
Ikeda et al (2022)	6-weeks old male mice (C57BL/6J): G1: PD + resveratrol monomer (NR) G2: PD + resveratrol dimer (NR)	Silk ligature around the maxillary left second molar during 15 days up to the end of experiment	At beginning of experiment	Intraperitoneal injection of a single dose of 10 mg/kg of body weight on day 15	Administration of resveratrol in dimeric or monomeric form reduced ABL. Resveratrol dimer induced better alveolar bone healing than resveratrol monomer. IL-1β was downregulated in the resveratrol dimer group.
Cirano et al (2021)	Adult male Wistar rats: G1: diabetes + placebo (13) G2: diabetes + insulin (14) G3: diabetes + R (13) G4: diabetes + R + insulin (14) G5: non diabetic + placebo (15)	Cotton ligature around the first mandibular molar and second maxillary molar during 11 days	19 days after the beginning of experiment	10 mg/kg of body weight 30 days (entire experiment) by gavage	Resveratrol reduced blood glucose, ABL and NADPH oxidase level. Resveratrol upregulated the level of mRNA for OPG and SIRT1.
Molez et al (2020)	Adult female Wistar rats: G1: ovariectomy + R (10) G2: ovariectomy + placebo (10) G3: ovariectomy + zoledronate + placebo (10) G4: ovariectomy + zoledronate + R (10) G5: non ovariectomy + placebo (10)	Cotton ligature around the first maxillary molar during 28 days	112 days after the beginning of experiment	10 mg/kg of body weight for 140 days (entire experiment) by gavage	Resveratrol reduced ABL and prevented bone density loss. Resveratrol reduced NADPH oxidase level.
Gimenez-Siurana et al (2020)	Male Sprague Dawley rats: G1: placebo (5) G2: CMC + silk fibroin nanoparticlues (5) G3: CMC + R (5) G4: CMC + silk fibroin nanoparticlues + R (5) G5: Water (5)	Ligature of silk thread around the mandibular incisors during 28 days	At beginning of experiment	1 ml of solution containing 3 mg/ml of resveratrol during 28 days (entire experiment) per os	Resveratrol had no influence on the inflammatory area. Resveratrol reduced gingival IL-6 and IL-1β.
Wagner et al (2019)	45 days old male Wistar rats: G1: PD + contrôle (10) G2: PD + red wine (10) G3: PD + grape juice (10) G4: PD + 12% alcohol (10) G5: PD + R (10)	Silk ligature around the second maxillary molar during 14 days	56 days after the beginning of experiment	0.05 mg/ml of resveratrol in the liquid. Liquid was available ad libitum	Resveratrol had no influence on ABL, gingival CRP, TNF-α or IL-6 level.
Correa et al (2019)	Adult male Wistar rats: G1: PD + smoke inhalation + placebo (13) G2: PD + smoke inhalation + R (13)	Cotton ligature around the first and second maxillary molars during 11 days	26 days after the beginning of experiment	10 mg/kg of body weight 30 days (entire experiment) by gavage	Resveratrol reduced ABL and increased tissue levels of SIRT and SOD and reduced levels of NADPH oxidase.
Correa et al (2018)	Adult male Wistar rats: G1: PD + arthritis induction + placebo (12) G2: PD + arthritis induction + ibuprofen (11) G3: PD + arthritis induction + R (11)	Cotton ligature on the mandibular first molars during 30 days	At beginning of experiment	10 mg/kg of body weight 30 days (entire experiment) by gavage	Resveratrol reduced ABL and increased serum IL-4 levels.
Ikeda et al (2018)	6–7 weeks old male mice (C57BL/6J): G1: PD + placebo (NR) G2: PD treatment + placebo (NR) G3: PD + R derivative-rich MSE (NR) G4: PD treatment + R derivative-rich MSE (NR)	Silk ligature around the maxillary left second molar during 15 days. In G2 and G4, ligatures were removed to simulate healing phase (treatment) while in G1 and G3 ligatures remained in place up to the end of experiment	At beginning of experiment	Intraperitoneal injection of a single dose 0.001% (w/w) of body weight on day 14	Resveratrol reduced ABL, gingival IL-1β level, and oxidative stress. Resveratrol down-regulated osteoclast formation and activity.
Correa et al (2017)	10 weeks old male Wistar rats: G1: PD + placebo (10) G2: PD + R (10) G3: PD + curcumin (10) G4: PD + curcumin + R (10)	Cotton ligature around one of the first mandibular molar during 30 days	At beginning of experiment	10 mg/kg of body weight 30 days (entire experiment) by gavage	Resveratrol reduced ABL and gingival IL-4 levels but not more than curcumin. Resveratrol reduced gingival IFN-g levels more than curcumin. Resveratrol associated with curcumin reduced gingival IL1-b level. Resveratrol and curcumin had no effect on gingival TNF-a level.
Ribeiro et al (2017)	10 weeks old male Wistar rats: G1: PD + smoke inhalation + R (20) G2: PD + smoke inhalation + placebo (20) G3: PD + placebo (20)	Cotton ligature around the first and second maxillary molars during 11 days	26 days after the beginning of experiment	10 mg/kg of body weight 30 days (entire experiment) by gavage	Resveratrol reduced ABL and prevented bone density loss. Resveratrol reduced gingival Th17/Th2 level, increased gingival IL-4 level, and downregulated RANKL gene expression.
Cirano et al (2016)	10 weeks old male Wistar rats: G1: PD + placebo (12) G2: PD + R (12)	Cotton ligature around one of the first mandibular molar during 11 days	19 days after the beginning of experiment	10 mg/kg of body weight 30 days (entire experiment) by gavage	Resveratrol treatment had no effect on Aa, Pg, and Tf concentrations in the cotton ligatures.
Chin et al (2016)	8 weeks old male Sprague Dawley rats: G1: control group (10) G2: PD + placebo (10) G3: PD + 0.1 mg/kg/d THSG (5) G4: PD + 10 mg/kg/d TSHG (5) G5: PD + 25 mg/kg/d R (5) G6: PD + 12.5 mg/kg/d of *P. mutiflora* ethanol extract (5) G7: PD + 25 mg/kg/d of *P. mutiflora* ethanol extract (5) G8: PD + 50 mg/kg/d of *P. mutiflora* ethanol extract (5)	Silk ligature around first mandibular molars during 8 days	1 day after the beginning of experiment	25 mg/kg of body weight 7 days (entire experiment) by gavage	Damage of periodontal bone in rats receiving 25 mg/kg resveratrol was reduced but there was no significant difference between resveratrol-treated group and control.
Bhattarai et al (2016)	Male Sprague Dawley rats: G1: control group (5) G2: PD + DMSO (5) G3: LPS + PD + DMSO (5) G4: LPS + PD + R (5)	Elastic ligature around the first and second maxillary molars during 14 days and some groups received 20 µl of 1 mg/ml LPS three times/week	At beginning of experiment	Subcutaneous injection of 5 mg/kg of body weight 14 days (entire experiment)	Resveratrol reduced ABL, formation of osteoclasts, and the production of circulating ROS. Resveratrol attenuated the production of COX-2, MMP-2 and MMP-9. Resveratrol increased bone mineral density and SOD activity. Resveratrol suppressed LPS-mediated decreases in HO-1 and Nrf2 levels in the inflamed periodontal tissues.
Zhen et al (2015)	6–8 weeks old C57BLKS/J male Mice: G1: control group (10) G2: PD + placebo (10) G3: PD + R (10)	Cotton ligature presoaked in medium containing P. gingivalis around the first maxillary molar during 28 days	At the beginning of experiment	20 mg/kg of body weight 28 days (entire experiment) by gavage	Resveratrol reduced levels of inflammatory cytokines (IL-1β, IL-6, IL-8, TNF-α) and TLR4 in gingival tissue. Resveratrol significantly lowered phosphorylation of NF-κB p65, p38MAPK, and STAT3, three transcription factors of TLR4.
Tamaki et al (2014)	8 weeks old male Wistar rats: G1: control group (6) G2: PD + placebo (6) G3: PD + R (6)	Ligature of thread around the right second maxillary molar during 20 days	At beginning of experiment	10 mg/kg of body weight 20 days (entire experiment) per os	Resveratrol reduced ABL and serum level of IL-6 and TNF-α. Resveratrol activated the Sirt1/AMPK and the Nrf2/antioxidant defense pathways and inhibited inflammatory Nf-kb/MAPk pathways.
Casati et al (2013)	10 weeks old male Wistar rats: G1: PD + placebo (12) G2: PD + R (12)	Cotton ligature around one of the first mandibular molar during 11 days	19 days after the beginning of experiment	10 mg/kg of body weight 30 days (entire experiment) by gavage	Resveratrol reduced ABL and gingival IL-17 level and tended to reduce gingival IL-1β level.
PD: Periodontal disease. R: Resveratrol. MSE: melinjo seed extract. G: group; NC: Not reported. Pg: Porphyromonas gingivalis. Aa: Aggregatibacter actinomycetemcomitans. Tf: Tannerella forsythia. ABL: Alveolar bone loss. OPG: Osteoprotegerin. Th: Helper T-cell. THSG: 2,3,5,4’-tetrahydroxystilbene-2-O-β-glucoside. DMSO: Dimethylsulfoxide. MMP: Matrix metalloproteinases. IL: Interleukin. TNF-α: Tumour necrosis factor-α. COX-2: Cyclooxygenase-2. SOD: Superoxide dismutase. NADPH: nicotinamide adenine dinucleotide phosphatase oxidase. SIRT1: Sirtuin 1. IFN-γ: Interferon-gamma. RANKL: Receptor activator of nuclear factor-kappa-B ligand. TLR: Toll-like receptor. HO-1: heme oxygenase-1. Nrf2: nuclear factor-E2 related factor 2. NF-κB : nuclear factor-κB. p38MAPK: p38 mitogen-activated protein kinase. STAT3: signal transducer and activator of transcription 3.					

Concerning the clinical studies presented in Table 2, all were randomised, double-blinded clinical trials. At the periodontal level, the patients presented different statuses according to the consensus report of workgroup 2 of the 2017 world workshop on the classification of periodontal and peri-implant diseases and conditions^30^: chronic periodontal disease (Stage II to Stage IV, grade B from the classification of Chicago 2017); moderate periodontal disease (Stage II); aggressive periodontal disease (Stage III–IV, grade C). Six control groups include a total of 143 participants, all of whom received a placebo as an adjuvant to periodontal therapy. The eight test groups include 215 patients who received resveratrol through various routes of administration. Three of the previously published studies involved non-surgical periodontal treatment combined with a single per os dose of resveratrol of 480 mg/d for 20 to 28 days.^20,28,43^ In the study of Zhang et al, the three test groups received different doses of resveratrol (125 mg/d, 200 mg/d, 500 mg/d) for 8 weeks, and no periodontal treatment was performed.^44^ The study by Mohammed et al evaluated a resveratrol-containing mouthwash used for 4 weeks alongside non-surgical periodontal therapy,^26^ whereas Lucchesi et al administered resveratrol systemically (500 mg/day for 180 days) in smokers with Stage III–IV grade C periodontitis, also combined with non-surgical therapy.^23^ Different clinical periodontal parameters such as plaque index (PI), clinical attachment level (CAL), bleeding index (BI), probing pocket depth (PPD), as well as inflammatory markers in serum, saliva, or gingival crevicular fluid (including IL-6, IL-1β, IL-10, TNF-α, IL-17, IL-33, IL-21, and IL-23), were recorded. None of these studies evaluated bone loss during follow-up, which varied from 4 weeks to 6 months depending on the trial.

**Table 2:** The main features of the selected clinical studies

Author (year of publication)	Patients included (sex)	Study design	Clinical and biochemical measurments	Groups (n/Group)	Characteristic of periodontitis	Treatment	Main outcomes
Mohammed et al (2025)	57 patients (33 males and 24 females)	RCT-DB	Primary outcomes: CAL, PPD, PI, BI Secondary outcomes: Salivary concentrations of IL-6 and RANKL.	1: placebo group (n = 20) 2: chlorhexidine group (n = 18) 3: resveratrol group (n = 19)	Chronic periodontal disease (Stage II or Stage III)	Resveratrol-containing mouthwash during 4 weeks + non-surgical treatment + instructions for dental hygiene	Resveratrol improved PI, BI, PPD but not CAL compared to placebo. Resveratrol reduced salivary concentration of IL-6 but not RANKL compared to placebo.
Lucchesi et al (2025)	38 smoking patients (15 males and 17 females)	RCT-DB	Primary outcomes: CAL Secondary outcomes: PGM, PI, BI, PPD, Gingival crevicular levels of IL-10, IL-17, IL-1β, IL-33, IL-21, IL-4, IL-23, IL-6, IFN-γ, and TNF-α	1: placebo group (n = 19) 2: resveratrol group (n = 19)	Chronic periodontal disease (Stage III or IV, grade C)	Resveratrol administration 500 mg/d during 180 days per os (capsule) + non-surgical treatment + instructions for dental hygiene	Resveratrol improved PPD, CAL, and PGM. Resveratrol reduced levels of IL-1β at 3 months and IL-6 at 3 months and 6 months in deep sites
Nikniaz et al (2023)	40 patients (17 males and 23 females)	RCT-DB	**Primary outcomes:** PPD, CAL **Secondary outcomes:** PI, BI, Salivary levels of IL-8 and IL-1β.	1: placebo group (n = 20) 2: resveratrol group (n = 20)	Chronic periodontal disease (Stage II to Stage IV, grade B)	Resveratrol administration 480 mg/d during 4 weeks per os (capsule) + non-surgical treatment + instructions for dental hygiene	Resveratrol improved PI. Resveratrol had no influence on the mean PPD, CAL, BI, and salivary levels of IL-8 and IL-1β.
Zhang et al (2022)	160 patients (72 males and 88 females)	RCT-DB	**Primary outcomes:** CAL, BI OHI-S, and PPD. **Secondary outcomes:** Serum and crevicular levels of TNF-a, GMCSF, MIP-1a, fibrinogen, CRP, INF-g, IL-2, IL-1b, IL-8, IL-4, IL-6, IL-10, IL-12p40, MCP1	1: placebo group (n = 40) 2: low-dose resveratrol group (n = 40) 3: middle-dose resveratrol group (n = 40) 4: high-dose resveratrol group (n = 40)	Agressive periodontal disease	Resveratrol administration 500 mg/d (high dose) or 200 mg/d (middle dose) or 125 mg/d (low dose) during 8 weeks per os (capsule)	Resveratrol improved CAL, BI OHI-S, and PPD. Resveratrol decreased all inflammatory markers in serum and crevicular fluid at all doses of resveratrol.
Javid et al (2019)	50 type 2 diabetic patients (NC)	RCT-DB	**Primary outcome:** CAL **Secondary outcomes:** Serum level of IL6, TNFa and TAC	1: placebo group (n = 22) 2: resveratrol group (n = 21)	Chronic periodontal disease	Resveratrol administration 480 mg/d during 28 days per os (capsule) + non-surgical treatment + instructions for dental hygiene	Resveratrol had no influence on TNF-α serum level, on TAC and CAL but reduced serum level of IL-6.
Javid et al (2017)	50 type 2 diabetic patients (36 females and 14 males)	RCT-DB	**Primary outcome:** PPD **Secondary outcomes:** Fasting blood glucose, insulin and triglyceride. Insulin resistance.	1: placebo group (n = 22) 2: resveratrol group (n = 21)	Moderate periodontal disease	Resveratrol administration 480 mg/d during 28 days (entire experiment) per os (capsule) + non surgical treatment + instructions for dental hygiene	Resveratrol treatment did not alter fasting blood glucose and triglyceride levels. However, resveratrol reduced insulin level, insulin resistance and PPD.
Abbreviations: y: year. IL: Interleukin. TNF-α: Tumour necrosis factor-α. PI: Plaque index. PPD: Probing pocket depth. CAL: Clinical attachment loss. BI: Bleeding index. PGM: Position of the gingival margin. OHI-S: Oral hygiene index-simplified. CRP: C-reactive protein, GMCSF: granulocyte-macrophage colony-stimulating factor, INF-g: interferon-gamma, MIP-1α: macrophage inflammatory protein-1alpha, MCP-1: monocyte chemoattractant protein-1. TAC: Total antioxidant capacity. RCT-DB: Randomised clinical trial – double-blinded.							

### Outcomes

Our primary outcome, ABL was only evaluated in 13 of the 16 selected preclinical studies.^3,7,9,10,12–14,17,18,27,35,39,41^ Except for one study, in which resveratrol did not show any effect^41^; a treatment with resveratrol attenuated from 7.09% to 60.60% alveolar bone loss parameter. Thus, the general analysis of preclinical studies has shown that resveratrol induces a reduction of ABL. Concerning secondary outcomes, our results show that resveratrol seems to improve some inflammatory parameters. For clinical studies, randomised controlled trials reveal more heterogeneous findings. Four studies reported clinical improvements, such as reductions in probing pocket depth (PPD), bleeding index (BI), plaque index (PI), and increases in clinical attachment level (CAL), following resveratrol administration.^23,26,43,44^ In contrast, two studies did not observe significant clinical improvements in patients with chronic periodontitis.^20,28^ Regarding biochemical markers, three studies demonstrated reductions in inflammatory cytokines – such as IL-6, IL-1β, or systemic endotoxin levels – in serum, saliva, or gingival crevicular fluid,^23,26,44^ whereas two studies reported no significant changes.^20,28^ As none of the clinical studies evaluated radiographic or clinical bone loss, the effects of resveratrol on ABL in humans cannot yet be established.

### Risk of Bias Within Studies

**Fig 2:**
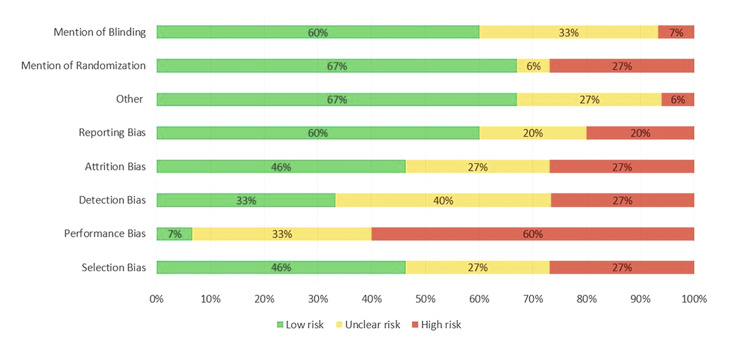
Risk of bias summary for preclinical animal studies. Risk of bias was assessed using SYRCLE’s Risk of Bias tool across the following domains: selection bias, performance bias, detection bias, attrition bias, reporting bias, and other sources of bias. Results are presented as percentages of studies rated as low, unclear, or high risk of bias for each domain. Additional descriptive items related to reporting quality (mention of randomisation and blinding) are presented separately and were not included in the formal risk of bias assessment. Green indicates low risk of bias, yellow indicates unclear risk of bias, and red indicates high risk of bias.

**Fig 3:**
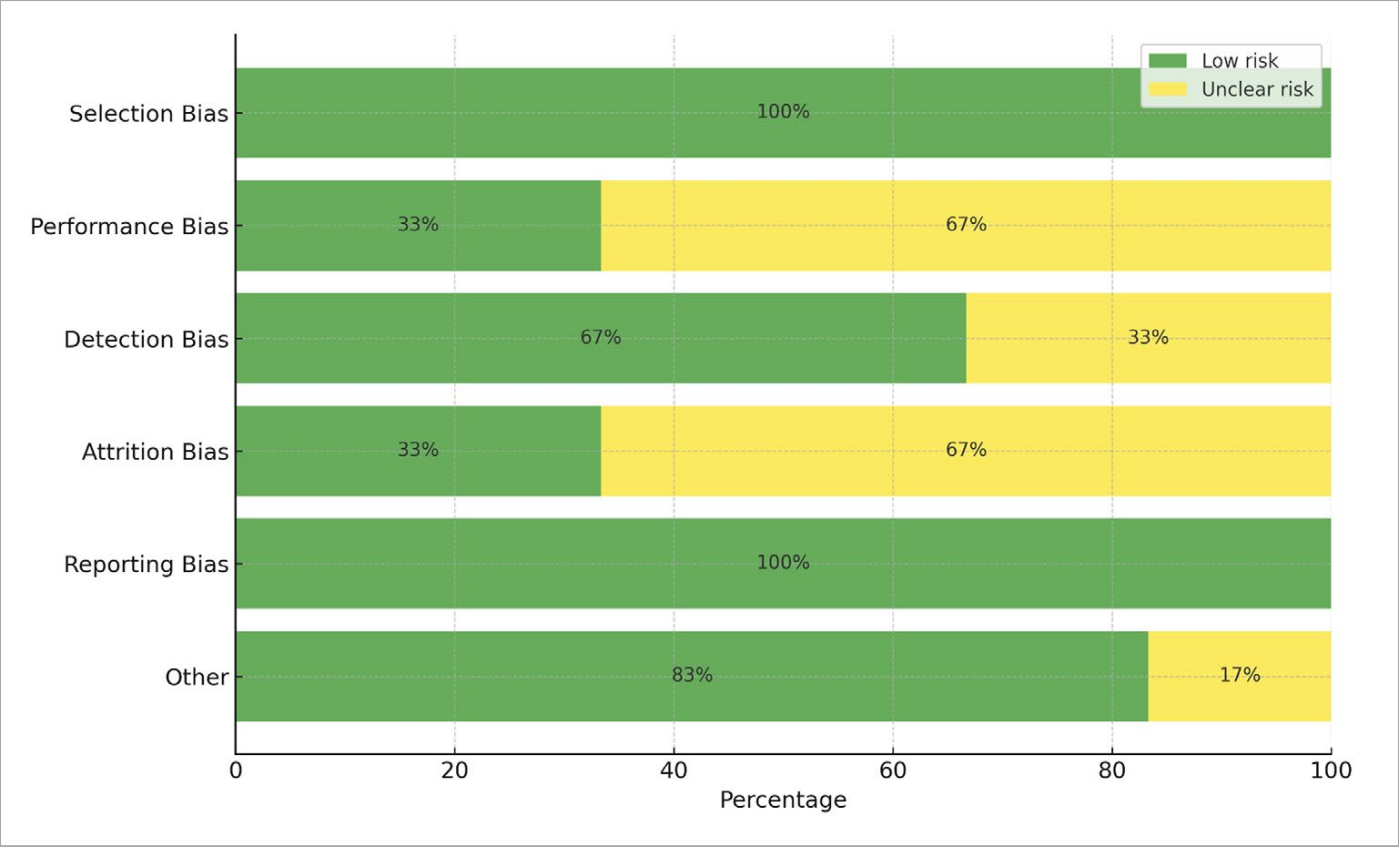
Risk of bias summary for clinical trials. Risk of bias was assessed using the Cochrane Risk of Bias tool, in accordance with the Cochrane Handbook. The following domains were evaluated: selection bias, performance bias, detection bias, attrition bias, reporting bias, and other bias. Results are expressed as percentages of studies classified as low or unclear risk of bias. Green indicates low risk of bias and yellow indicates unclear risk of bias.

Figures 2 and 3 summarise the results of the risk-of-bias assessment for preclinical animal studies and randomised controlled trials, respectively, as described in the Methods and Materials section. Overall, preclinical studies showed a predominantly unclear to high risk of bias across several domains, whereas randomised trials demonstrated a lower risk profile but with some domains remaining unclear. For preclinical studies, a substantial proportion of articles did not clearly report key methodological details, resulting in a high proportion of unclear risk for selection, performance, and detection bias (27%, 33%, and 40%, respectively). Notably, performance bias showed the highest proportion of high risk (60%), while detection bias also presented concerns (27% high risk). Attrition bias was rated as low risk in 46% of studies, and most studies were considered at low risk of reporting bias (60%). For randomised controlled trials, no study was classified as having a high risk of bias. However, performance and attrition bias remain the most frequent concerns, with 67% of the included studies presenting an unclear risk in these domains. Detection bias also shows some variability, with 33% of studies assessed as having an unclear risk. In contrast, all studies are considered to have a low risk of bias for selection and reporting, and 83% present a low risk regarding other potential sources of bias.

## DISCUSSION

This systematic review examines the conflicting results on the efficacy of resveratrol in controlling and treating periodontitis through preclinical animal models and human studies. Out of 22 identified studies, it was found impossible to conduct a meta-analysis due to variations in study design, models used, resveratrol treatment protocols, methods of inducing periodontitis, and measured outcome parameters. These differences make direct comparison of results challenging, thus necessitating a descriptive and systematic analysis to synthesise the available information.

### Bone Protection Effect of Resveratrol

Alveolar bone loss (ABL) is a critical clinical symptom of periodontitis, driven primarily by osteoclasts, the cells responsible for bone resorption. Given that controlling ABL is a major goal in periodontal disease treatment, numerous preclinical studies have investigated the effects of resveratrol on bone loss. In eight preclinical studies with similar animal models and resveratrol dose, reductions in ABL varied from 7.09% to 28.58% in animals treated with resveratrol.^7,11–14, 27,35,39^

Bhattarai et al and Ikeda et al found that resveratrol inhibited osteoclast formation,^3,17^ while Ribeiro et al reported that resveratrol could reduce receptor activator of nuclear factor-kappa B Ligand (RANKL) gene expression.^35^ RANKL activates osteoclasts by binding to the receptor activator of nuclear factor-kappa B (RANK), triggering the process of bone resorption.

Despite the promising findings, two studies included in our systematic review reported no significant improvement in ABL in animals treated with resveratrol. However, in one study, the treatment period was only seven days,^9^ and in the other one the resveratrol dose (0.05 mg/ml) was likely lower than in other studies.^41^ These findings suggest that the effectiveness of resveratrol may be influenced by the duration and dosage of treatment. More studies are needed to determine the optimal dose and treatment duration required to mitigate ABL.

### Anti-inflammatory Effect of Resveratrol

During the inflammatory process associated with periodontitis, there is a significant increase in the expression of pro-inflammatory cytokines, such as IL-1β, IL-6, and transforming growth factor-β (TGF-β), which play key roles in the disease’s progression.^33^ The anti-inflammatory properties of resveratrol have been extensively studied, attracting significant attention from the scientific community.^24^

Our systematic review revealed that numerous preclinical studies reported reductions in levels of IL-1β, IL-6, IL-8, and TNF-α in the gingival tissues of mice or rats treated with resveratrol. These cytokines are not only important markers of inflammation but also play functional roles in the progression of periodontal tissue destruction and the development of other long-term complications. IL-1β and TNF-α are known to promote osteoclast activity,^21^ thereby contributing to periodontal breakdown. Resveratrol appears to exert its anti-inflammatory effect by reducing the activation of the transcription factor NF-κB.^2^

### Anti-Oxidant Effect of Resveratrol

Oxidative stress is widely recognised as a key factor in the development of inflammatory conditions like periodontitis. During periodontitis, immune cells release reactive oxygen species (ROS) and inflammatory cytokines to combat periodontal pathogens. However, these molecules also play a significant role in the aetiology of local tissue damage through complex interactions between periodontal pathogens and the host’s immune response. Notably, nitric oxide pathways are increasingly recognised as key regulators of both oxidative stress and antimicrobial activity in periodontal tissues.^36,37^

Resveratrol has been identified as a potent antioxidant. For instance, Corrêa et al demonstrated that resveratrol treatment reduces nicotinamide adenine dinucleotide phosphate hydrogen (NADPH) oxidase levels, even in the presence of cigarette smoke inhalation.^12^ Other studies have similarly shown that resveratrol regulates NADPH oxidase expression, an enzymatic complex responsible for the formation of the initial ROS, which then leads to the production of other ROS. NADPH oxidase subunits, known as NOX (NOX 1–5), catalyse the reduction of oxygen to superoxide using NADPH as an electron donor.^5^ These NOX enzymes are significantly elevated in periodontal tissues during periodontitis.^34^

### Clinical Studies

To date, only six clinical studies on the use of resveratrol in the treatment of periodontal disease have been published. Resveratrol shows greater efficacy in aggressive periodontitis (Stage III–IV, grade C) than in chronic periodontitis, likely due to its anti-inflammatory and antioxidant properties. However, its clinical use is limited by small sample sizes, methodological heterogeneity, and lack of long-term data. Since resveratrol is typically administered orally in human clinical trials, its efficacy may be compromised due to lower bioavailability compared to animal studies. However, the analysis of available data on oral bioavailability in humans is hindered by the methodological inconsistencies prevalent in existing literature.^38^ However, we showed that resveratrol can inhibit periodontal bone loss when administered orally in animals. Therefore, the route of administration may not be critically important, as biological effects have been observed with various delivery methods, including oral administration. Nonetheless, resveratrol bioavailability could be different between humans and animals, and it would be interesting to conduct further studies using resveratrol in topical applications in humans.

### Clinical Relevance

In terms of clinical relevance, the findings of this review suggest that resveratrol may be considered as a promising adjunct to nonsurgical periodontal therapy, particularly due to its anti-inflammatory and antioxidant properties. The available clinical studies indicate potential improvements in key periodontal parameters such as probing pocket depth, bleeding index, and clinical attachment level when resveratrol is used alongside conventional scaling and root planing. These effects may be especially relevant in patients presenting with heightened inflammatory burden or systemic conditions associated with oxidative stress, such as diabetes or smoking. However, the current evidence remains limited by small sample sizes, heterogeneity in study design, and variability in dosage and administration routes, which precludes the establishment of standardised clinical protocols. In contrast, no direct evidence supports the use of resveratrol in surgical periodontal therapy, as none of the included studies specifically evaluated its impact in regenerative or surgical contexts. Therefore, while resveratrol appears to be a potentially valuable adjunct in nonsurgical management of periodontitis, further well-designed randomised controlled trials are required to determine its efficacy, optimal delivery methods, and possible role in surgical periodontal interventions.

## CONCLUSION

This systematic review underscores the potential of resveratrol as an adjunctive therapy for periodontitis, particularly in preclinical models. While clinical trials show promising anti-inflammatory effects, further research is needed to optimise dosage, duration, and delivery methods, and to evaluate its impact on alveolar bone loss in humans.

### Acknowledgements

The authors also gratefully acknowledge the organisation ParoNantes.

#### Funding

No funding was received for this study.

#### Availability of data and materials

Not applicable to this article as no data sets were generated or analysed.

#### Ethics approval and consent to participate

Not applicable; this systematic review, utilising publicly available information, does not require ethics approval.

## References

[ref1] Andrade EF, Orlando DR, Araujo AMS, de Andrade J, Azzi DV, de Lima RR (2019). Can resveratrol treatment control the progression of induced periodontal disease? A systematic review and meta-analysis of preclinical studies. Nutrients.

[ref2] Ben Lagha A, Andrian E, Grenier D (2019). Resveratrol attenuates the pathogenic and inflammatory properties of Porphyromonas gingivalis. Mol Oral Microbiol.

[ref3] Bhattarai G, Poudel SB, Kook SH, Lee JC (2016). Resveratrol prevents alveolar bone loss in an experimental rat model of periodontitis. Acta Biomater.

[ref5] Brown DI, Griendling KK (2009). Nox proteins in signal transduction. Free Radic Biol Med.

[ref6] Bunte K, Hensel A, Beikler T (2019). Polyphenols in the prevention and treatment of periodontal disease: a systematic review of in vivo, ex vivo and in vitro studies. Fitoterapia.

[ref8] Cekici A, Kantarci A, Hasturk H, Van Dyke TE (2014). Inflammatory and immune pathways in the pathogenesis of periodontal disease. Periodontol 2000.

[ref9] Chin YT, Hsieh MT, Lin CY, Kuo PJ, Yang YC, Shih YJ (2016). 2,3,5,4’-tetrahydroxystilbene-2-O-beta-glucoside isolated from Polygoni Multiflori ameliorates the development of periodontitis. Mediators Inflamm.

[ref10] Cirano FR, Casarin RC, Ribeiro FV, Casati MZ, Pimentel SP, Taiete T (2016). Effect of resveratrol on periodontal pathogens during experimental periodontitis in rats. Braz Oral Res.

[ref11] Cirano FR, Molez AM, Ribeiro FV, Tenenbaum HC, Casati MZ, Correa MG (2021). Resveratrol and insulin association reduced alveolar bone loss and produced an antioxidant effect in diabetic rats. J Periodontol.

[ref12] Correa MG, Absy S, Tenenbaum H, Ribeiro FV, Cirano FR, Casati MZ (2019). Resveratrol attenuates oxidative stress during experimental periodontitis in rats exposed to cigarette smoke inhalation. J Periodontal Res.

[ref13] Correa MG, Pires PR, Ribeiro FV, Pimentel SP, Cirano FR, Napimoga MH (2018). Systemic treatment with resveratrol reduces the progression of experimental periodontitis and arthritis in rats. PLoS One.

[ref14] Correa MG, Pires PR, Ribeiro FV, Pimentel SZ, Casarin RC, Cirano FR (2017). Systemic treatment with resveratrol and/or curcumin reduces the progression of experimental periodontitis in rats. J Periodontal Res.

[ref15] Galiniak S, Aebisher D, Bartusik-Aebisher D (2019). Health benefits of resveratrol administration. Acta Biochim Pol.

[ref16] Gimenez-Siurana A, Gomez Garcia F, Pagan Bernabeu A, Lozano-Perez AA, Aznar-Cervantes SD, Cenis JL (2020). Chemoprevention of experimental periodontitis in diabetic rats with silk fibroin nanoparticles loaded with resveratrol. Antioxidants (Basel).

[ref17] Ikeda E, Ikeda Y, Wang Y, Fine N, Sheikh Z, Viniegra A (2018). Resveratrol derivative-rich melinjo seed extract induces healing in a murine model of established periodontitis. J Periodontol.

[ref18] Ikeda E, Tanaka D, Glogauer M, Tenenbaum HC, Ikeda Y (2022). Healing effects of monomer and dimer resveratrol in a mouse periodontitis model. BMC Oral Health.

[ref19] Inchingolo AD, Inchingolo AM, Malcangi G, Avantario P, Azzollini D, Buongiorno S (2022). Effects of resveratrol, curcumin and quercetin supplementation on bone metabolism – a systematic review. Nutrients.

[ref20] Javid AZ, Hormoznejad R, Yousefimanesh HA, Haghighi-Zadeh MH, Zakerkish M (2019). Impact of resveratrol supplementation on inflammatory, antioxidant, and periodontal markers in type 2 diabetic patients with chronic periodontitis. Diabetes Metab Syndr.

[ref21] Kanno Y (2024). The roles of fibrinolytic factors in bone destruction caused by inflammation. Cells.

[ref22] Krayer JW, Leite RS, Kirkwood KL (2010). Non-surgical chemotherapeutic treatment strategies for the management of periodontal diseases. Dent Clin North Am.

[ref23] Lucchesi VH, Giorgetti APO, Corrêa MG, Pecorari VGA, Benatti BB, Tenenbaum HC (2025). Impact of systemic resveratrol on non-surgical periodontal treatment of smokers: a 12-month randomized clinical trial. Clin Oral Investig.

[ref24] Meng T, Xiao D, Muhammed A, Deng J, Chen L, He J (2021). Anti-inflammatory action and mechanisms of resveratrol. Molecules.

[ref25] Mizutani K, Buranasin P, Mikami R, Takeda K, Kido D, Watanabe K (2021). Effects of antioxidant in adjunct with periodontal therapy in patients with type 2 diabetes: a systematic review and meta-analysis. Antioxidants (Basel).

[ref26] Mohammed SA, Akram HM (2025). Evaluating the efficacy of resveratrol-containing mouthwash as an adjunct treatment for periodontitis: a randomized clinical trial. Eur J Dent.

[ref27] Molez AM, do Nascimento EHL, Haiter Neto F, Cirano FR, Pimentel SP, Ribeiro FV (2020). Effect of resveratrol on the progression of experimental periodontitis in an ovariectomized rat model of osteoporosis: morphometric, immune-enzymatic, and gene expression analysis. J Periodontal Res.

[ref28] Nikniaz S, Vaziri F, Mansouri R (2023). Impact of resveratrol supplementation on clinical parameters and inflammatory markers in patients with chronic periodontitis: a randomized clinical trail. BMC Oral Health.

[ref31] Park HJ, Jeong SK, Kim SR, Bae SK, Kim WS, Jin SD (2009). Resveratrol inhibits Porphyromonas gingivalis lipopolysaccharide-induced endothelial adhesion molecule expression by suppressing NF-kappaB activation. Arch Pharm Res.

[ref32] Pihlstrom BL, Michalowicz BS, Johnson NW (2005). Periodontal diseases. Lancet.

[ref33] Ramadan DE, Hariyani N, Indrawati R, Ridwan RD, Diyatri I (2020). Cytokines and chemokines in periodontitis. Eur J Dent.

[ref34] Rausch-Fan X, Matejka M (2001). From plaque formation to periodontal disease, is there a role for nitric oxide. Eur J Clin Invest.

[ref35] Ribeiro FV, Pino DS, Franck FC, Benatti BB, Tenenbaum H, Davies JE (2017). Resveratrol inhibits periodontitis-related bone loss in rats subjected to cigarette smoke inhalation. J Periodontol.

[ref37] Shang J, Liu H, Zheng Y, Zhang Z (2023). Role of oxidative stress in the relationship between periodontitis and systemic diseases. Front Physiol.

[ref38] Szymkowiak I, Marcinkowska J, Kucinska M, Regulski M, Murias M (2025). Resveratrol bioavailability after oral administration: a meta-analysis of clinical trial data. Phytother Res.

[ref39] Tamaki N, Cristina Orihuela-Campos R, Inagaki Y, Fukui M, Nagata T, Ito HO (2014). Resveratrol improves oxidative stress and prevents the progression of periodontitis via the activation of the Sirt1/AMPK and the Nrf2/antioxidant defense pathways in a rat periodontitis model. Free Radic Biol Med.

[ref40] Trindade D, Carvalho R, Machado V, Chambrone L, Mendes JJ, Botelho J (2023). Prevalence of periodontitis in dentate people between 2011 and 2020: a systematic review and meta-analysis of epidemiological studies. J Clin Periodontol.

[ref41] Wagner MC, Cavagni J, Gaio EJ, Brum VS, Jesus LH, Filho MS (2019). Effect of red wine and its major components on periodontitis and systemic inflammation in rats. J Int Acad Periodontol.

[ref42] Yahfoufi N, Alsadi N, Jambi M, Matar C (2018). The immunomodulatory and anti-inflammatory role of polyphenols. Nutrients.

[ref43] Zare Javid A, Hormoznejad R, Yousefimanesh HA, Zakerkish M, Haghighi-Zadeh MH, Dehghan P (2017). The impact of resveratrol supplementation on blood glucose, insulin, insulin resistance, triglyceride, and periodontal markers in type 2 diabetic patients with chronic periodontitis. Phytother Res.

[ref44] Zhang Q, Xu S, Xu W, Zhou Y, Luan H, Wang D (2022). Resveratrol decreases local inflammatory markers and systemic endotoxin in patients with aggressive periodontitis. Medicine (Baltimore).

[ref45] Zhen L, Fan DS, Zhang Y, Cao XM, Wang LM (2015). Resveratrol ameliorates experimental periodontitis in diabetic mice through negative regulation of TLR4 signaling. Acta Pharmacol Sin.

